# Prenatal ketamine exposure causes abnormal development of prefrontal cortex in rat

**DOI:** 10.1038/srep26865

**Published:** 2016-05-26

**Authors:** Tianyun Zhao, Chuanxiang Li, Wei Wei, Haixing Zhang, Daqing Ma, Xingrong Song, Libing Zhou

**Affiliations:** 1 Department of Anesthesiology, Guangzhou Women and Children’s Medical Center, Guangzhou Medical University, Guangzhou, PR China; 2 Department of Anesthesiology, Third Affiliated Hospital of Southern Medical University, Guangzhou, PR China; 3 Anaesthetics, Pain Medicine & Intensive Care, Department of Surgery & Cancer, Faculty of Medicine, Imperial College London, Chelsea and Westminster Hospital, London, UK; 4 Guangdong-Hongkong-Macau Institute of CNS Regeneration, Ministry of Education CNS Regeneration Collaborative Joint Laboratory, Jinan University, Guangzhou, PR China; 5 Co-innovation Center of Neuroregeneration, Nantong University, Jiangsu, PR China

## Abstract

Ketamine is commonly used for anesthesia and as a recreational drug. In pregnant users, a potential neurotoxicity in offspring has been noted. Our previous work demonstrated that ketamine exposure of pregnant rats induces affective disorders and cognitive impairments in offspring. As the prefrontal cortex (PFC) is critically involved in emotional and cognitive processes, here we studied whether maternal ketamine exposure influences the development of the PFC in offspring. Pregnant rats on gestational day 14 were treated with ketamine at a sedative dose for 2 hrs, and pups were studied at postnatal day 0 (P0) or P30. We found that maternal ketamine exposure resulted in cell apoptosis and neuronal loss in fetal brain. Upon ketamine exposure *in utero*, PFC neurons at P30 showed more dendritic branching, while cultured neurons from P0 PFC extended shorter neurites than controls. In addition, maternal ketamine exposure postponed the switch of NR2B/2A expression, and perturbed pre- and postsynaptic protein expression in the PFC. These data suggest that prenatal ketamine exposure impairs neuronal development of the PFC, which may be associated with abnormal behavior in offsprings.

Ketamine is widely used clinically for anesthesia, especially in the developing world^1^. Up to 2% pregnant women require surgery, due to problems associated with pregnancy itself or to other medical conditions, and ketamine a common choice. This number is increasing, partly because of laparoscopic procedures and fetal surgery^2^. Furthermore, ketamine is a popular drug of abuse in diverse populations including pregnant women in the West^3^,^4^ and Southeast Asia^5^. As ketamine may have a prolonged effect on the central nervous system, it is important to investigate whether its use in pregnant subjects may affect neurodevelopment of their offspring.

General anesthetics may be toxic to the developing brain^6^,^7^,^8^,^9^, as evidenced by increased apoptotic cells, impaired neurogenesis, and neuroinflammation^10^. In rodents and primates, some anesthetics used in early-life might impair cognitive function, an effect that can last into adulthood^11^,^12^. These findings are supported by retrospective clinical data which indicate that the use of anesthetics during early life may be associated with late-onset learning disabilities^8^,^13^. Learning and memory function may not be the sole target of toxicity associated with anesthetics, and emotional disorders such as depression and anxiety have not been studied.

We previously reported that^14^, maternal exposure to ketamine for 2 hrs on gestational day 14 induced anxiety and depression-like behavior^15^, and cognitive impairment in offsprings. The mismatched expression of *N*-methyl-*D*-aspartate (NMDA) receptor subunits and decreased levels of brain-derived neurotrophic factor and postsynaptic density-95 protein (PSD-95) may be responsible for the abnormal behavior^14^. Emotion modulates memory and learning, interferes with decision making and provides the motivation for critical action^16^. Conversely, cognitive processing is an integral part of emotion: Anxiety and depression lead to information processing biases and cognitive dysfunction, which contribute to the onset and/or maintenance of symptoms^17^. Cognition and emotion are intricately intertwined, and both are implemented by overlapping networks in brain regions including the PFC, hippocampus and amygdala^18^. The hippocampus to PFC pathway plays a critical role in learning and memory^19^. The PFC receives input from all other cortical regions and hippocampus, and mediates planning and goal-directed motor, cognitive, and affective behavior^20^. Compared to other areas, the maturation of the PFC is protracted and therefore more susceptible to harmful factors, contributing to neuropsychiatric disorders^21^. As the abnormal development of the PFC is responsible for diverse emotional, cognitive, and mnemonic problems^22^, we tested whether the maternal exposure to ketamine impacts the development of PFC, and explored some potential mechanisms.

At low and subanesthetic doses, ketamine is a selective and potent antagonist of NMDA receptors^23^. In the brain, NMDA receptors are involved in neuronal development and circuit formation by mediating synaptic activity, organizing functional circuits and regulating dendritic growth^24^,^25^. Two NMDA receptor subunits, NR2A and NR2B, have specific effects during dendritic arbor development, and a switch in expression of NR2B to NR2A occurs during synaptogenesis^25^,^26^. The cytoplasmic C-terminus of NR2A and NR2B interacts with membrane-associated guanylate kinase members and PSD-95, which is essential for clustering and anchoring NMDA receptors at synapses^27^,^28^. In this study, we aimed to test the hypothesis that maternal ketamine exposure may disturb the temporal switch of NR2A/2B subunits and impact dendritic development via the PSD-95/NR2A/2B-NMDA receptors in the PFC.

## Results

### Ketamine induces a widespread apoptosis in fetal brain

**Figure 1:**
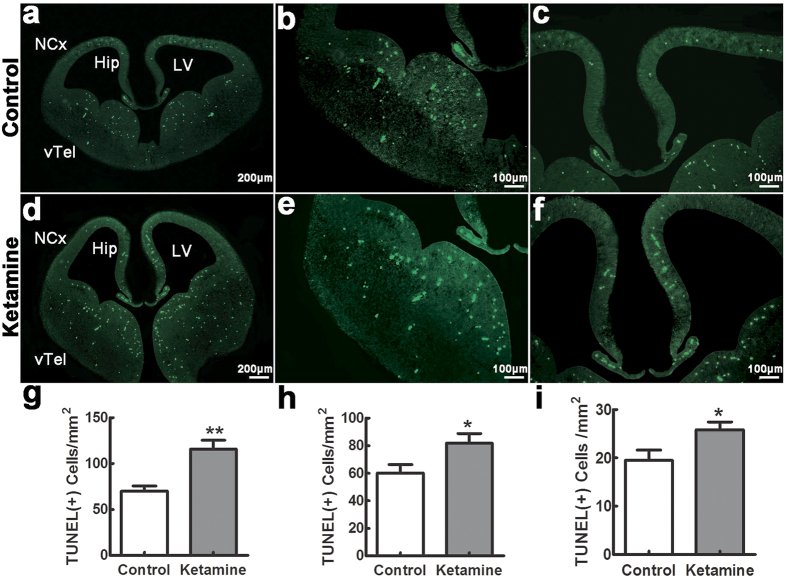
Ketamine induces a widespread apoptosis in fetal brain. Embryonic day 14 coronal sections were stained with TUNEL. In control (**a–c**) and ketamine (**d–f**) groups, positive cells were widely distributed in the ventral telencephalon (vTel), hippocampal primordium (Hip) and neocortex (NCx). Positive cells in different brain areas were significantly increased in the ketamine compared to the control group (**g**): vTel; (**h**): Hip; (**i**): NCx). LV, lateral ventricle. *p < 0.05; **p < 0.01; n = 6 in each group.

**Figure 2:**
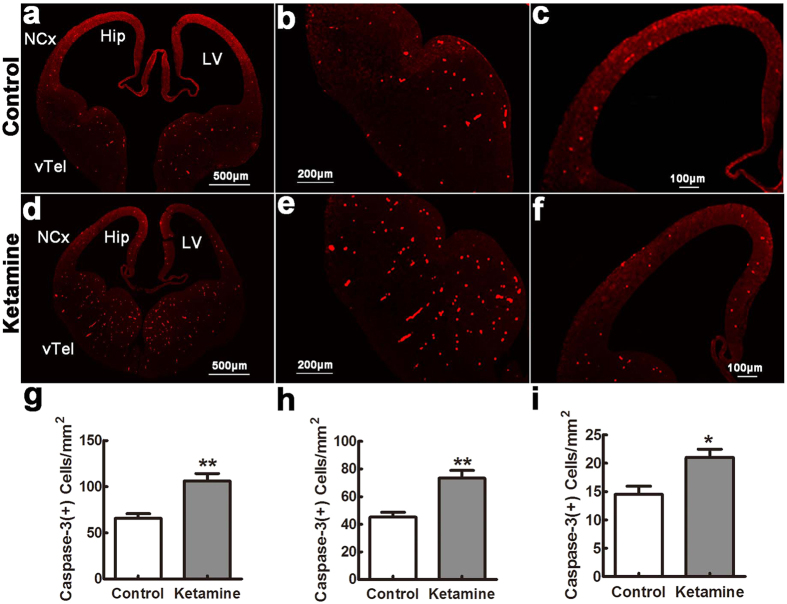
Caspase-3 positive cells in the fetal brain. Embryonic day 14 sections were stained with antibodies against cleaved caspase-3. In control (**a–c**) and ketamine (**d–f**) groups, positive cells were widely distributed in the ventral telencephalon (vTel), hippocampal primordium (Hip) and neocortex (NCx). Their densities were significantly increased in the ketamine compared to the control group in different areas ((**g**) vTel; (**h**) Hip; (**i**) NCx). LV, lateral ventricle. *p < 0.05; **p < 0.01; n = 6 in each group.

To assess apoptosis, TUNEL-positive cells were counted in different regions 8 hrs after ketamine administration and in control fetuses. The number of TUNEL positive cells was significantly larger in the ketamine than in the control group (Fig. 1), particularly in the ventral telencephalon (Fig. 1b,e,g, 116 ± 9 cells/mm^2^ vs. 70 ± 6 cells/mm^2^, *t* = 4.199, *p* = 0.001), hippocampal primordium (Fig. 1c,f,h, 82 ± 7 cells/mm^2^ vs. 60 ± 6 cells/mm^2^, *t* = 2.319, *p* = 0.039) and neocortex (Fig. 1a,d,i, 26 ± 2 cells/mm^2^ vs. 20 ± 2 cells/mm^2^, *t* = 2.381, *p* = 0.039). As necrotic cells also contain fragmented DNA and TUNEL cannot distinguish between apoptosis and necrosis, we also used anti-activated caspase-3 immunofluorescence to detect apoptotic profiles. We found that positive cells were significantly increased in the ketamine compared to the control group (Fig. 2), in the ventral telencephalon (Fig. 2b,e,g, 106 ± 8 cells/mm^2^ vs. 66 ± 5 cells/mm^2^, *t* = 4.257, *p* = 0.001), hippocampus primordium (Fig. 2c,f,h, 73 ± 6 cells/mm^2^ vs. 45 ± 4 cells/mm^2^, *t* = 4.224, *p* = 0.001) and neocortex (Fig. 2c,f,i, 21 ± 2 cells/mm^2^ vs. 15 ± 1 cells/mm^2^, *t* = 3.153, *p* = 0.011).

### Ketamine causes neuronal loss in laminae II-III of the PFC in offspring

**Figure 3:**
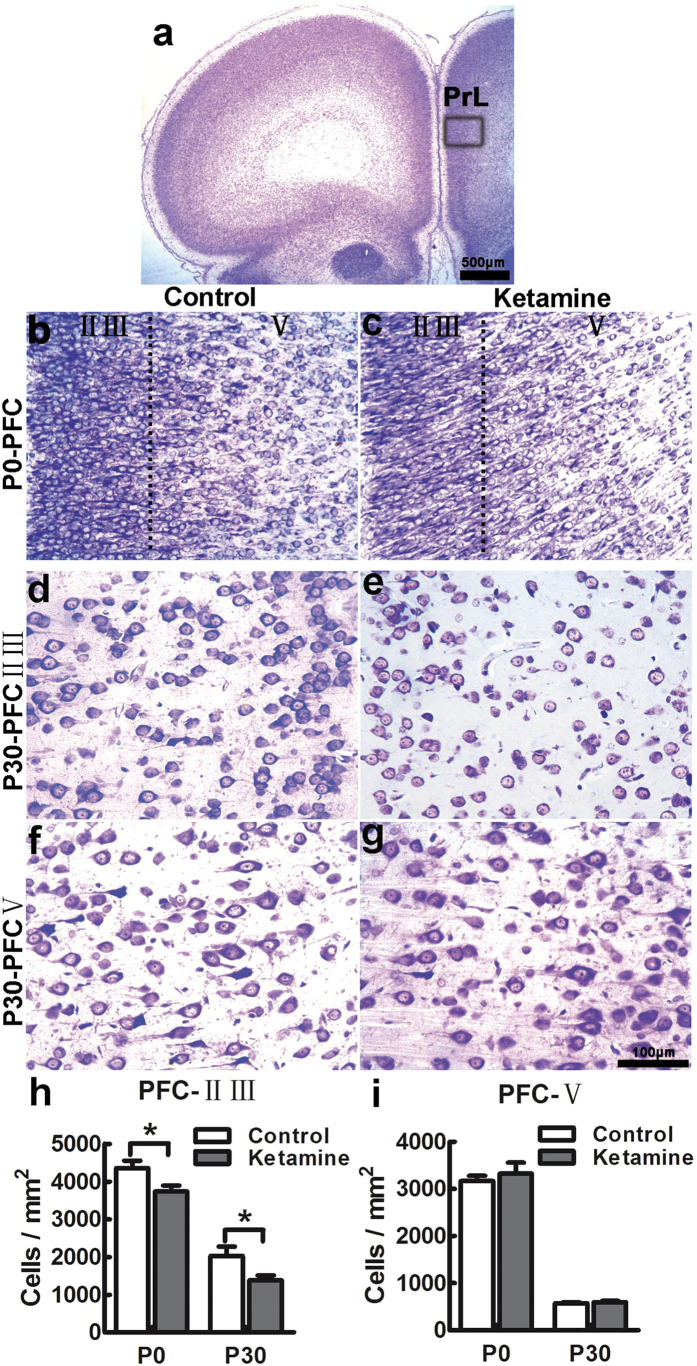
Ketamine causes neuronal loss in laminae II-III of the PFC in offspring. Coronal sections encompassing the PFC at P0 and P30, stained with cresyl violet. The boxed area indicated in panel a was chosen for analysis. At P0, the cytoarchitecture of the PFC was comparable in control (**b**) and ketamine (**c**) groups, but the cell density in laminae II-III was significantly decreased in the ketamine group (**h**). At P30, cell distribution was similar in laminae II-III (**d,e**) and V (**f,g**) of control (**d,f**) and ketamine-treated animals (**e,g**). In the ketamine group, the cell density was significantly decreased in laminae II-III (**h**) but not in lamina V (**i**), compared to the control group. PrL, prelimbic cortex; *p < 0.05; n = 6 in each group.

We previously found that ketamine-treated offsprings developed mental disorders in adulthood^14^. As the PFC is a key hub in neural networks that modulate emotional behavior^29^, we studied the architecture of the PFC at P0 and P30 using Nissl staining. Compared to the control group, cell density in laminae II-III of ketamine-treated offsprings was decreased by 14% at P0, and 32% at P30 (Fig. 3b–e,h; *t* = 2.354, *p* = 0.040 and *t* = 2.302, *p* = 0.044, respectively). However, there was no significant difference in cell density in lamina V of the PFC at P0 and P30 (Fig. 3b,c,f,g,i, *t* = 0.628, *p* = 0.544 and *t* = 0.645, *p* = 0.533, respectively). Thus, neurons in laminae II-III appear more susceptible to ketamine.

### Ketamine exposure disturbs the maturation of pyramidal neurons in the PFC of offspring

**Figure 4:**
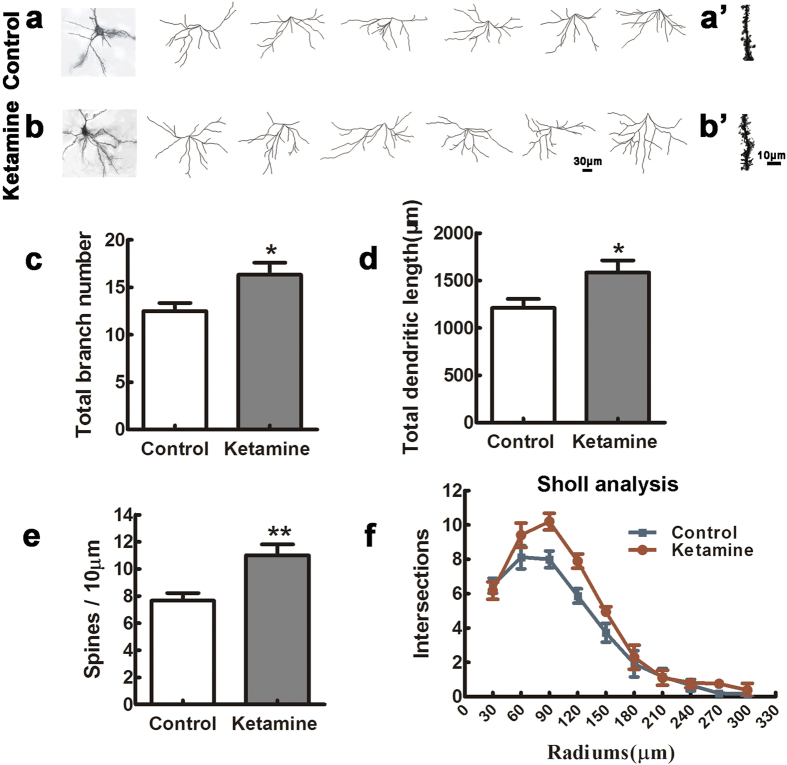
Ketamine exposure disturbs the maturation of pyramidal neurons in the PFC of offspring. Six P30 brains in each group were processed for Golgi–Cox impregnation and pyramidal neurons in laminae II-III of the PFC were studied in the control (examples in a) and ketamine (examples in b) groups. The left panels of (**a,b**) show two examples of Golgi-impregnated neurons. The total branch number and dendritic length were significantly increased in the ketamine compared to the control group (**c,d**) n = 60 neurons in each group). In addition, the ketamine group had higher spine density than the control (a’, b’, (**e**) n = 60 dendrites in each group). Sholl analysis showed that the complexity of dendritic trees was higher in the ketamine than in the control group (**f**) Kolmogorov–Smirnov test). *p < 0.05; **p < 0.01.

To study pyramidal neurons, we performed Golgi–Cox impregnation and reconstructed basal and apical dendritic trees of 120 pyramidal neurons in each group (10 neurons per region and 6 pups per group) selected from laminae II-III and V of the PFC. Basal and apical dendrites, and the total dendritic branch length in neurons of lamina V were similar in the ketamine and control groups (data not shown). In contrast, in laminae II-III, neurons from ketamine-treated pups had more basal branches (Fig. 4a–c, n = 6, *t* = 2.495, *p* = 0.032) and longer total branch length than in the control group (Fig. 4a,b,d, *t* = 2.370, *p* = 0.039). This was confirmed by Sholl analysis (Fig. 4f, *p* = 0.009). Furthermore, the spine density was significantly increased in laminae II and III neurons from ketamine-treated offsprings compared with that in control samples (Fig. 4a’, *t* = 3.371, *p* = 0.007).

### Ketamine administration inhibits neurite growth in neuronal culture

**Figure 5:**
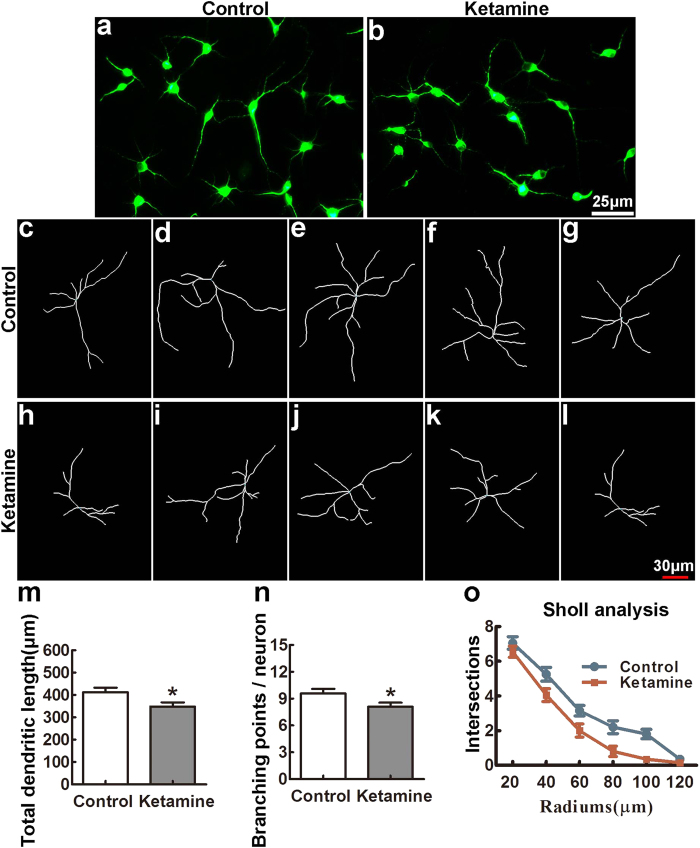
Ketamine administration *in utero* inhibits neurite growth in neuronal culture. P0 PFC neurons from pups whose mothers received ketamine at gestational day 14 or not (control) (n = 6 in each group) were cultured for 4 DIV, immunostained with anti-*β*-tubulin antibodies (**a,b**), prior to reconstruction using Imaris (examples in (**c–g**), control; (**h–l**), ketamine group). Quantitative analysis showed that the total neurite length and number were significantly lower in the ketamine than the control groups ((**m,n**); n = 60 neurons in each group). This difference was confirmed by Sholl analysis (**o**). *p < 0.05; **p < 0.01.

To verify that anomalies of pyramidal neurons in animals exposed to ketamine *in utero* are due to a cell intrinsic defect, we cultured PFC neurons from control and ketamine-treated P0 pups. As shown in Fig. 5, after 4 days *in vitro* (DIV), neurons from both groups were comparable in their ability to extend neurites. However, quantitative analysis showed that the total neurite length was significantly shorter and the branching was significantly decreased in the ketamine-treated compared to the control group (Fig. 5m,n, *t* = 2.389, *p* = 0.020 and *t* = 2.246, *p* = 0.029, respectively). Similar results were obtained by Sholl analysis (Fig. 5o, *p* < 0.01). These data suggested that maternal administration of ketamine resulted in a cell autonomous decrease in neurite extension of PFC neurons *in vitro.*

### Expression of NR2B/NR2A, synaptophysin and PSD-95 is affected by maternal exposure of ketamine

**Figure 6:**
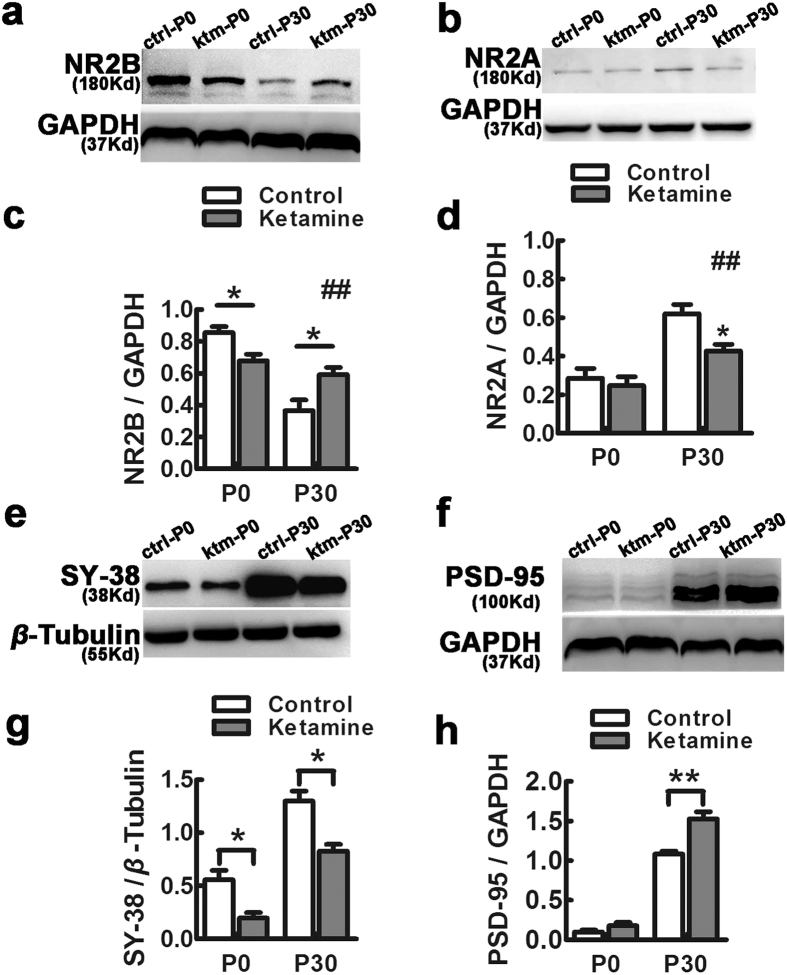
Expression of NR2B/2A, PSD-95 and synaptophysin is affected by maternal exposure to ketamine. PFC tissue from P0 and P30 rats were processed for western blots to detect proteins NR2B (**a**), NR2A (**b**), synaptophysin (SY-38, e) and PSD-95 (**f**). The NR2B protein levels in the ketamine group were significantly lower at P0 and higher at P30 compared to the corresponding control samples (**a,c**). The NR2A protein was expressed at a low level at P0 in both control and ketamine samples (**b,d**) but was significantly decreased in the ketamine compared to the control at P30 (**b,d**). The ketamine group expressed significantly less SY-38 at P0 and P30 relatively to controls (**e,g**). In contrast, there was no significant difference of PSD-95 levels at P0 and more PSD-95 protein in the ketamine group than in the control at P30 (**f,h**). ctrl-P0, control group at P0; ctrl-P30, control group at P30; ktm-P0, the ketamine group at P0; ktm-P30, ketamine group at P30. *p < 0.05; **p < 0.01, comparison between the control and ketamine group; ^#^*p* < 0.05; ^##^*p* < 0.01, comparison between P0 and P30; n = 6 in each group.

**Figure 7:**
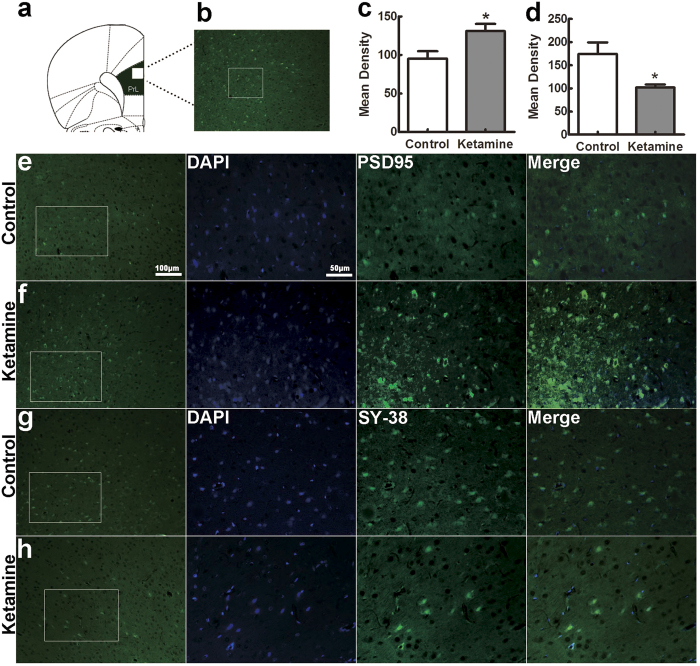
The numbers of PSD-95 and synaptophysin positive cells in PFC are modified by ketamine exposure. The rat with location of the medial prefrontal cortex (PrL, **a**). Areas of prefrontal cortex as indicated in (**b**) and laminae II-III as indicated in (**e–h**) were selected for analysis. Paraffin coronal sections from P30 PFC were stained with anti-PSD-95 and anti-synatophysin (SY-38) antibodies. Fluorescent immunohistochemistry for PSD-95 (green) and SY-38 (green), and DAPI (blue). The signal was estimated by quantifying the density of immnofluorescence using Image J. The ketamine group showed a significant increase of PSD-95 immunoreactivity (**c**) and a significant decrease of synaptophysin (**d**) at P30 compared to the control. *p < 0.05; n = 6 in each group.

We measured the expression of NR2B and NR2A, the presynaptic protein synaptophysin and the postsynaptic protein PSD-95 in the PFC at P0 and P30 (Fig. 6). The expression of NR2B was significantly higher in the control than the ketamine-treated group at P0 (Fig. 6a,c, 0.857 ± 0.038 vs. 0.680 ± 0.040, *F* = 10.24, *p* = 0.033). In contrast, the situation was different at P30, when NR2B levels were slightly lower in the ketamine than in the control group (Fig. 6a,c, the control vs. the ketamine: 0.367 ± 0.066 vs. 0.593 ± 0.044, *F* = 8.260, *p* = 0.045). NR2A protein was expressed a low level at P0, with no significant difference in the two groups (Fig. 6b,d, 0.287 ± 0.050 in control vs. 0.247 ± 0.046 in ketamine, *F* = 0.346, *p* = 0.588). Interestingly, the NR2A protein level was significantly decreased in the ketamine compared to the control at P30 (Fig. 6b,d, ketamine vs. control: 0.427 ± 0.035 vs. 0.620 ± 0.047, *F* = 10.745, *p* = 0.031). The level of synaptophysin was significantly higher in the control than the ketamine-treated group at both P0 and P30 (Fig. 6e,g, *t* = 3.590, *p* = 0.023 and *t* = 2.874, *p* = 0.014, respectively). In contrast, PSD-95 was weakly and similarly expressed in both groups at P0, but very significantly higher in ketamine-treated rats than control animals at P30 (Fig. 6f,h, *t* = 4.732, *p* = 0.009). In addition, to better define the distribution and localization of synaptic proteins, we performed double immunofluorescence using anti-PSD-95 and anti-synaptophysin (SY-38) antibodies on PFC sections from P30 brains. In accord with the results obtained with western blot, there was clearly higher fluorescence intensity for PSD-95 and less immunoreactivity of synaptophysin in the ketamine group compared to the control (Fig. 7c,d, *t* = 2.661, *p* = 0.024 and *t* = 2.859, *p* = 0.017, respectively).

## Discussion

The present study provides evidence that maternal administration of subanesthetic dose of ketamine during pregnancy in rats induces apoptosis and neuronal loss in fetal brain, as well as abnormalities of PFC layer II/III pyramidal neuron development. In the PFC, there are mismatched expression of NMDA receptor subunits and disturbed expression of pre- and postsynaptic proteins at P0 and P30. Together with our previous study, these results indicate that *in utero* ketamine exposure has detrimental effects on brain development, particularly in the hippocampus and PFC.

Anesthetics can be toxic for the developing brain, and two critical factors determine neurotoxicity: the stage of brain development at which the compound is administered, and the dose and/or duration of exposure^30^. Neurodevelopment proceeds at a different pace in different brain regions, so that vulnerability depends both on stage and region. Although the dose in our experiment was subanesthetic, sedative dose^23^ could induce cell apoptosis in fetal brain. This is consistent with a previous observation that ketamine exposure of fetal rhesus monkeys resulted in 2.2 times more neuronal loss than exposure at early postnatal stages^6^. Our data confirm that fetal brains are more vulnerable to ketamine than neonatal ones, with preferential neuronal loss in laminae II and III of PFC region. We previously showed that ketamine administration *in utero* impaired postnatal neurogenesis in the dentate gyrus and in the subventricular zone. It is therefore likely that ketamine induced neuronal loss by impacting the proliferation and/or apoptosis of neural progenitor cells, which remains to be demonstrated further.

The hippocampus and PFC play well-established roles in cognitive and mnemonic processes. Psychiatric disorders, such as schizophrenia, depression and post-traumatic stress syndrome, share common symptoms of cognitive impairment and emotional dysregulation and are associated with structural and pathophysiological changes in the hippocampus and PFC^31^. Our previous study showed that ketamine exposure impaired maturation of pyramidal cells in the CA3 hippocampal field of offspring (at P30)^14^. In contrast, the present study shows that pyramidal neurons in laminae II and III of the PFC of offspring (at P30) harbor the opposite profiles, exhibiting more branched and longer basilar dendrites, and increased spine density after ketamine exposure. This discrepancy is likely due to regional differences of anaesthesia-induced neurotoxic effect on synaptogenesis and neuroplasticity^32^. Moreover, our data are consistent with reports that psychoactive drugs such as amphetamine, cocaine or nicotine, increased spine density in PFC and nucleus accumbens^33^,^34^. For layer II/III cells, basal dendrites receive inputs from local sources and apical dendrites receive inputs from other cortical areas as well as nonspecific thalamic inputs^35^,^36^. Our results indicates that exposure of ketamine *in utero* may influence local synaptic circuits in PFC in adulthood.

In addition to inducing apoptosis, anesthetics interfere with synaptogenesis^37^,^38^. Most excitatory inputs are targeted to dendrites and the establishment of dendritic trees is a highly dynamic process characterized by extension and retraction of branches, followed by their stabilization and establishment of synaptic connections^39^. We investigated dendritic development at two developmental time points, early (cultured PFC neurons at P0) and mature stages (P30, Golgi staining). In cultured neurons derived from neonatal PFC, the total neurite length was significantly shorter and neurites less ramified in the ketamine than in the control group. This is consistent with a previous observations that ketamine induced neuronal damage in rat primary forebrain cultures^40^. Intriguingly, our *in vivo* data at P30 showed an opposite over-branched dendritic phenotype upon ketamine exposure. A possible explanation is that PFC neurons at P0 are at an early stage of dendritic development, characterized by dynamic branch extension, and this may be dampened by ketamine treatment. In contrast, neurons at P30 are at the end of those dynamic processes, when the overall effect of ketamine could result in dendritic over-branching. Given that Golgi impregnation does not work well at P0, other techniques, such as electron microscopy and electrophysiology-coupled imaging are required to test that hypothesis.

NMDA receptors are important for neuronal development and circuit formation^24^. The NR2B subunit is highly expressed in the embryonic and neonatal brain, whereas expression of the NR2A subunit increases during brain maturation^41^. This ‘developmental switch’ of the subunit content of synaptic NMDA receptors plays a key role in refining topographic projections, organizing functional circuits and regulating dendritic growth^26^. In control animals, we found that NR2B was abundantly expressed at P0 and significantly decreased at P30, while NR2A was expressed at a low level at P0 and significantly increased at P30, as reported^42^. However, the reduction of NR2B protein at P30 and the increase of NR2A expression were not as prominent in ketamine treated neurons. This blunted ‘developmental switch’ of NR2B/NR2A expression induced by ketamine may cause the impaired dendritic development of PFC neurons, and may be involved in the behavioral phenotype in offsprings. NMDA receptors are predominantly expressed at the postsynaptic membrane where they interact with components of the postsynaptic density that modulate their activity, particularly PSD-95^43^. PSD-95 regulates the size and strength of synapses^44^, the formation of synaptic assemblies^45^, and spine-maturation^46^. Synaptophysin is one of the most abundant synaptic vesicle integral proteins that regulates the formation and trafficking of synaptic vesicles^47^. Our Western blot and immunohistochemistry data showed that the expression of PSD-95 and synaptophysin at P0 and P30 was similar to what was reported elsewhere^48^,^49^. The higher levels of PSD-95 and NR2B expression in ketamine-treated than in the control samples presumably reflects increased synapse concentration, which is consistent with the overbranched dendritic phenotype in mature neurons^39^. The decreased expression of synaptophysin at P0 and P30 may reflect abnormal transmitter release in ketamine-treated offsprings.

In summary, together with our previous study^14^, the present results show that exposure to ketamine *in utero* can impair the development of the hippocampus and the PFC, both of which might contribute to cognitive impairment and mood disorders in offspring. This information has important implications for the use of ketamine in anesthesia and as a recreational drug, especially during pregnancy.

## Materials and Methods

### Subjects

All experimental procedures were performed according to the guidelines that have been approved by the Ethics Committees of Jinan University, Guangzhou, China. The Sprague-Dawley rats, weighted 180–220 g, were housed with a 12 hr light/dark cycle and had access to water and food *ad libitum*. The vaginal plugs were checked in the morning and a positive one was defined as the gestational day 0. Ketamine injections were done on gestational day 14.

### Anesthesia

**Figure 8:**
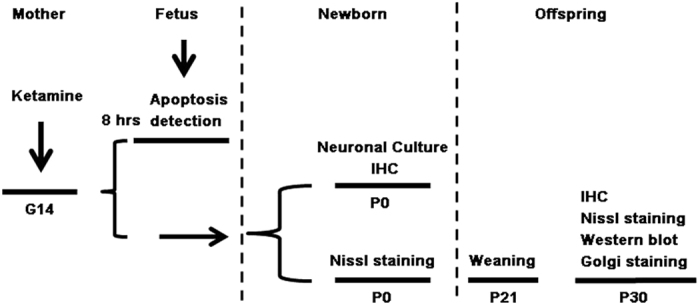
The flow chart of the experimental protocols.

On gestational day 14, 18 dams were randomly divided into control (n = 9) and ketamine (n = 9) groups. The establishment of animal models was described before^14^. Three dams per group were used for TUNEL assay 8 hrs after ketamine infusion whilst another six in each group were allowed to give birth naturally. Three P0 pups from each dam were used for neuronal culture and each experiment was repeated six times. P0 and P30 brains were used for histology and western blot. Golgi stain was carried out on P30 brains. A schematic representation of experimental protocols is shown in Fig. 8. The total dose of ketamine used in each dam was 144.2 ± 4.6 mg/kg (n = 9), and all anesthetized dams recovered fully without complications, such as respiratory depression, cardiac arrest, or miscarriage. The recovery time from anesthesia was 37 ± 4 min (n = 9), and the total anesthesia time (from initial intramuscular injection to recovery) was 176 ± 4 min. At P30, the body weight of rats in the ketamine group was less than that of the control group (71 ± 3 g vs. 88 ± 3 g; *t* = 3.770, *p* = 0.0003).

### Histology and immunohistochemistry

Ten paraffin coronal sections (5 μm) including the PFC (+2.7 mm from bregma and +3.7 mm from interaural landmark) were stained with 0.5% cresyl violet (n = 6 in each pup). Microphotographs were taken under a 40× objective (Leica, DM6000B, Germany). Nissl-stained cell numbers were counted in a blinded manner using Image J. (NIH, USA). For Immunohistochemistry, the sections were incubated with the following primary antibodies overnight at 4 °C: goat anti-cleaved caspase-3 antibody (L-18) (1:250, Cat.No.sc-1225, Santa Cruz, CA, Germany), mouse anti-Synaptophysin (1:1000, Cat.No.ab8049, Abcam, Cambridge, UK), mouse- anti-PSD-95 (1:1000, Cat.No. MAB1596, Millipore, USA). Signal was detected with Alexafluor 546- or 488-labeled fluorescent secondary antibodies (1:1000, Invitrogen, Carlsbad, CA, USA). Caspase-3 positive cells and PSD-95 and synaptophysin immunofluorescence intensity were evaluated using Image J (NIH, USA).

### TUNEL analysis

Eight hrs after ketamine infusion, embryonic brains were fixed in 4% paraformaldehyde (PFA) overnight at 4 °C, then processed and embedded in wax. Ten to fifteen paraffin coronal sections (5 μm) of each brain (n = 6, 2 from each dam) were stained with a TUNEL kit (C1086, Beyotime institute of Biotechnology, China). Positive cells were examined under the microscope with a digital camera system (Leica).

### Golgi stain

Golgi stain, tri-dimensional reconstruction and dendrite analysis were performed as described^14^. Frozen brain sections with 150 μm-thickness between +2.7 mm from bregma and +3.7 mm from interaural landmark were stained using a FD Rapid Golgi Stain kit (FD NeuroTechnologies, Inc. Columbia, USA) and 10 well individualized pyramidal neurons in superficial (II–III) and deep (V) laminaes were reconstructed using with the Imaris software (BitPlane AG, Zurich, Switzerland). To measure spine density, distal segments of dendrites of pyramidal neurons selected for reconstruction were photographed using a 100× objective for counting of spines in 40 μm segments, and the results were expressed as the number of spines /10 μm.

### Neuronal cultures and neurite analysis

Cerebral cortices from PFC were chopped into pieces and digested with 0.25% trypsin (w/v) in HBSS at 37 °C for 30 min. The reaction was stopped by fetal bovine serum (Cat.No.10099133, Gibco). Samples were triturated by gentle pipetting and cells were harvested by centrifugation at 1000 rpm for 5 min, and seeded on polylysine-coated coverslips at a density of 1.0 × 10^5^ cells/ml. The culture medium was neurobasal-A medium (Cat.No.10888022, Invitrogen) supplemented with 2% B27 (Cat.No.17504044, Invitrogen). After 4 DIV, cells were fixed with 4% PFA and immunostained with rabbit anti-*β*-tubulin (1:1000, Cat.No.2128, Cell Signaling Technology). The images of 10 well developed neurons were captured and neurites were analyzed using the Imaris software (BitPlane AG). The complexity of total dendritic trees was estimated using Sholl analysis^50^.

### Western blotting

Samples from PFC (n = 6/group) were dissociated in lysis buffer (containing protease inhibitors, 50 mM Tris–HCl, pH 7.6) on ice for 30 min and homogenized via ultrasonification (Ningbo scientz biotechnology CO. LTD, Ningbo, China). After centrifugation at 12,000 g for 10 min at 4 °C, supernatants were pooled and protein concentrations were measured with a BCA assay kit (Beyotime Institute of Biotechnology, China). Thirty μg protein samples were mixed with gel loading buffer (50 mM Tris-HCl, 10% SDS, 10% glycerol, 10% 2-mercaptoethanol, 2 mg/ml bromophenol blue) in a 1:1 ratio, boiled for 5 min and subjected to SDS-PAGE gels. The separated proteins were transferred to polyvinylidene fluoride membranes and incubated with mouse anti-Synaptophysin (1:1000, Cat.No.ab8049, Abcam, Cambridge, UK), rabbit anti-NR2B (1:1000, Cat.No.06-600, Millipore, USA), rabbit anti-NR2A (1:1000, Cat.No.4205, Cell Signaling Technology, USA), mouse anti-PSD-95 (1:1000, Cat.No.MAB1596, Millipore, USA), rabbit anti-GAPDH (1:1000, Cat.No.5174, Cell Signaling Technology, USA) and rabbit anti-*β*-tubulin (1:1000, Cat.No.2128, Cell Signaling Technology, USA) overnight at 4 °C. Signal was detected using HRP-conjugated rabbit or mouse antibodies followed by chemiluminescence using a Super Signal West Pico kit (Pierce) and Hyper film ECL (Amersham Biosciences). All experiments were carried out at least in triplicate. Autoradiography films were scanned and signals were quantified using Image J (NIH, USA). Density of each band was normalized to reference controls (*β*-tubulin or GAPDH).

### Statistical analysis

Data were presented as mean ± SEM. The homogeneity of variance was verified with Levene’s test and single comparison between both groups was made using unpaired two-tailed Student *t* test. Data on expression of NR2B and NR2A were analyzed by two-way ANOVA, and significant main effects of two different timepoints were followed by one-way ANOVA for each group. The parametric Bonferroni and nonparametric Kruskal–Wallis H tests were used to analyze dendrite branching with the Sholl method. Results were considered significant (* or ^#^) at *p* < 0.05 and highly significant (** or ^# #^) at *p* < 0.01. All analyses were performed using SPSS 16.0 software (SPSS Inc., Chicago, IL, USA).

## Additional Information

**How to cite this article**: Zhao, T. *et al.* Prenatal ketamine exposure causes abnormal development of prefrontal cortex in rat. *Sci. Rep.* **6**, 26865; doi: 10.1038/srep26865 (2016).
